# Economics: the view from below

**DOI:** 10.1186/s41937-017-0019-2

**Published:** 2018-01-25

**Authors:** Marion Fourcade

**Affiliations:** University of California-Berkeley, Berkeley, CA USA

There is a long tradition among sociologists of using economics as an intellectual straw man. For a while in the 1980s and 1990s, every economic sociology article, on every topic, ran something like this: it began by ritualistically presenting the view from economics, and then proceeded to pull it to pieces by showing that “it is, in fact, much more complicated” than the play of interests and incentives. Against what they regarded as naïve economism, sociologists and anthropologists “demonstrated” the relevance of social networks, or culture, or politics to explaining various economic outcomes, and questioned the standard of “economic rationality.” They celebrated their “dirty hands” against the economists’ “clean models” and worried about the encroachments of rational choice theory into their discipline (Hirsch et al. 1987; Wacquant and Calhoun 1989). To the “anthropological monster” that they saw in Homo Economicus, and in Stigler and Becker’s injunction that “De Gustibus non est disputandum”, they opposed their own sociological theories of action, rooted in a fine-grained understanding of how necessity shapes choice and how relative social position shapes taste (Bourdieu 2000).

Paradoxically perhaps -but in what amounts to a classic defense of the disciplinary boundary- the sociologists’ main targets in those days were often those economists closest to them intellectually: the economic historian Alfred Chandler, the institutionalists Oliver Williamson and Douglas North; and the microeconomist Gary Becker (who had a joint appointment in the Chicago sociology department).^1^ Economists, of course, did not care very much about all of this agitation on the other side of the disciplinary border. Most were probably unaware of the sociologists’ anxious fixation with their science. And those who knew about it, like Gary Becker, possibly felt: never mind, all publicity is good publicity.^2^

The fixation, eventually, gave way to something a bit deeper and hopefully more productive: first, sociologists (and others, such as historians and science studies scholars) started to free themselves from their inferiority complex and became more confident in their own contribution to the analysis of economic processes (some of which, like network analysis, has influenced recent economic research); second, they turned their analytic lens toward economics itself. They started to investigate the sources of the economists’ authority and its complicated relationship to democratic politics; building on the contributions of historians of economics, they probed the discipline’s development over time and its variability across nations, shattering the myth of a universal science; and they strove to make sense of what the expansion of economic technique means for the way we live our lives. This is what this talk aims to share: a view of economics from its outside, looking at it from below.

## The veiled moralists

In the course of the twentieth century, economists have been able to establish a remarkable position for themselves, as experts in local and national governmental organizations, in independent agencies and central banks, in international institutions, in business and finance, and in the media. They supplanted lawyers in government and historians in the public sphere. As such, they have been involved with some of the most consequential decisions that societies make—decisions having to do, for instance, with the level of unemployment that might be left unattended, because it should be considered “natural”; with whether or not to authorize the purchase and sale of untested financial products or with how to organize the delivery of clean water, vaccines or electricity.

This involvement has come at a cost. As Robert Chernomas and Ian Hudson put it, “economics has the awkward distinction of being both the most influential and the most reviled social science” (2016, 3). We might add: economics may be the most reviled social science *precisely because* it is the most influential. First, for better or for worse, economics has become the science of making social choices, and as such it always cuts right through the heart of politics and morality. The acknowledgement that (to quote Karl Marx) “[political economy] is—for all its worldly and debauched appearance—a truly moral science, the most moral science of all” (2005, 361) would have been no surprise to a long line of distinguished economic thinkers, from the classical economists to the founders of welfare economics, from John Maynard Keynes (who saw economics as a branch of ethics) to Tony Atkinson, who recently reminded the field that economists *constantly* enounce normative judgments (Atkinson 2009, also see Randazzo and Haidt 2015). Strangely enough, however, consideration of the normative hypotheses and criteria that underlie these judgments is often brushed aside as inconsequential. It is not. Economists tend to, well, economize, and societies often resist. Applying cost-benefit analysis to a host of social problems might increase efficiency, but it might also create or reinforce social inequalities in access to goods and services. It might crowd out moral norms, work ethics, and civic ideals. Certain proposed solutions (like value-added models to calculate teachers’ compensation, a recent fad in education) could quite simply feel distasteful and divisive. So the moral debate is, in fact, inevitable—and perhaps should be. As philosopher Michael Sandel (2013, 138) put it in the pages of the *Journal of Economic Perspectives*, “The more economic thinking extends its reach into social and civic life, the more market reasoning becomes inseparable from moral reasoning.”

## The fortune tellers

The second reason why economists may feel particularly reviled is more mundane: The public is quick to trace failures in any policy area back to “some academic scribbler of a few years back” (as Keynes put it in a famous quote). This blame game is lopsided: the counterfactual does not exist—that is, the public often does not know which crises might have been averted thanks to some academic scribbler a few years back. So the failures stand out more than the successes. Non-economist intellectuals and marginal scholars within economics are only too happy to double down on the criticism of the mainstream: It gives them a jolt of *Shadenfreude*, and the opportunity to momentarily shine in the sun.

All of these sentiments were, of course, painfully exposed in the aftermath of the 2008 financial crisis, which threw the entire field of macroeconomics into a deep, soul-searching exercise and prompted a hunt for culprits, from regulators asleep at the wheel to high profile academics too comfortably cozied up to Wall Street. But what was most shocking to the profession, perhaps, was that the episode had been almost entirely unforeseen by the vast majority of its members, including those who claimed expertise in the areas of finance and macroeconomics (Caballero 2010). It was a powerful reminder that spectacular historical failures to predict the economic future have always been an essential part of the profession’s history, often buoyed by the belief that *this* bubble is not a bubble, and the notion that “this time is different” (Reinhart and Rogoff 2011). Sadly, what is never different is the extraordinary vulnerability of economic prediction to long tail events and the always humbling test of reality. (I should say, as a side note, that this vulnerability is true of *all* social scientific knowledge, not just economics. Just recall political science’s and sociology’s abysmal failure to predict the collapse of communism, or the Arab spring, or the recent wave of populist elections in the West.)

Importantly, elite status does not protect from public embarrassment; if anything it intensifies it, by commanding increased publicity. In *The Inside Job*, the filmmaker Charles Ferguson piercingly documented the blind sightedness, and occasionally the sheer dishonesty, of several high profile members of the economics profession in the run-up to the US financial crash. The situation seemed to echo the 1930s, not just on the economic, but also on the intellectual front. Two days before Black Thursday in October 1929 the perhaps most distinguished American economist of his time and the head of what was then one of the country’s most prominent economic forecasting businesses, Yale professor Irving Fisher, was quoted in the *New York Times* as saying that “stock prices are low.” Fisher persisted in his optimism for months after the crash, as the economy (and his reputation) spiraled downwards (Friedman 2014, 82–83). But he was not alone in being humiliated by facts. In 1929, the Harvard Economic Society, Fisher’s main competitor service (with ties to the Harvard’s economics department), did not see the Great Depression coming either (Friedman 2014, 157–159)^3^.

Of course the susceptibility of individual experts—especially the most reputable and influential ones—to error matters a lot in practice. It may not be too far-fetched to suggest that Irving Fisher and the Harvard Economic Service’s belief that stocks were undervalued counted among the factors that fueled the speculation boom of 1927 and 1928. The close involvement of two Nobel laureates, Robert Merton and Myron Scholes, in Long Term Capital Management may have allowed the hedge fund to obtain far more credit than it could handle—one cause of its spectacular failure in 1998. Enthusiastic or benign expert reports by academics about the Icelandic or Irish economies may have played a role in masking real vulnerabilities. And as William Easterly (2014) has suggested repeatedly, the idealism of many development economists (Jeffrey Sachs claiming that we are nearing “the end of poverty”) has been possibly drawing attention and resources away from the most enduring basic problems. The list goes on.

## The pretense of knowledge

This, however, is a rather superficial critique. A more serious one might look beyond the influence of specific (and misguided) individuals, toward the broader conditions that made those individuals and their social position possible. 1929, 1998 and 2008 exposed what Friedrich Hayek, in his 1974 Nobel lecture, chastised as economics’ “pretense of knowledge.” (Hayek 1989) In Hayek’s view, the pretense of knowledge derives from two kinds of illusion: first, the idea that the social world, which is made of “phenomena of unorganized complexity,” can be known. The fundamental problem of economic order, Hayek had been arguing since the 1930s, is the fact that knowledge (what we call today “information”) is dispersed (Hayek 1945); second, the notion that such knowledge can be attained through measurement and measurement only. “While in the physical sciences the investigator will be able to measure what, on the basis of a prima facie theory, he thinks important, in the social sciences often is treated as important that which happens to be accessible to measurement. This is sometimes carried to the point where it is demanded that our theories must be formulated in such terms that they refer only to measurable magnitudes. It can hardly be denied that such a demand quite arbitrarily limits the facts which are to be admitted as possible causes of the events which occur in the real world” (1989, 3). Modern science studies scholars would not disagree with Hayek’s skepticism about statistics: we often take the “measurable magnitudes” as given—and yet they are anything but. How we slice up the economic world, count and refuse to count, or aggregate, are contingent and evolving historical conventions, nothing more (e.g. see Stapleford 2009). Change the convention, as the proponents of the Human Development Index have suggested for instance, and the picture of economic reality changes, too—sometimes dramatically.

Echoing Hayek’s critique, many scholars closer to us have noted that economics’ remarkable emphasis on analytical rigor, elegance and formal conventionalism, their aspirations to “micro-theory-like precision” are often obtained at the expense of a close adherence to facts, and at the risk of irrelevance (Caballero 2010, 100; also McCloskey 1985, Mayer 1993, Krugman 2009, Romer 2015). Through ever-finer precision in measurement and mathematics, economists have constructed a wholly separate and artificial reality. It is this new reality—not the world as it is, but a “scholastic illusion” of it (Bourdieu 2000)—that shapes the ever more sophisticated internal games toward which all subsequent practitioners orient themselves and in which they seek to make their mark. As Ricardo Caballero noted in a remarkably candid commentary on macroeconomics after the financial crisis “by some strange herding process the core of macroeconomics seems to transform things that may have been useful modeling short-cuts into a part of a new and artificial “reality,” and now suddenly everyone uses the same language, which in the next iteration gets confused with, and eventually replaces, reality. Along the way, this *make-believe substitution* raises our presumption of knowledge about the workings of a complex economy and increases the risks of a pretense of knowledge about which Hayek warned us” (2010, 89, my emphasis)^4^.

Hayek, however, went even further. Economics’ “propensity to imitate as closely as possible the procedures of the brilliantly successful physical sciences” (1989, 3), he argued, has sustained an over-optimistic “striving to control society” (1989, 7), “as if one needed only to follow some cooking recipes to solve all social problems” (1989, 6).^5^ The time (we were in 1974) seemed ripe for such a critique. Stagflation was taking hold across many Western economies and, Hayek warned, “we have at the moment little cause for pride: as a profession we have made a mess of things.” Hayek was not simply calling into question the discipline’s faulty predictive ability (the Irving Fischer problem): he was actually accusing his colleagues of *causing* the (unforeseen) economic mess in the first place.

Obviously Hayek—being Hayek—had in mind a very particular “mess of things.” He was referencing economists’ propensity to intervene in the world, particularly their foolish (or so he thought) belief that one can achieve full employment by manipulating aggregate demand. That belief, of course, had come out of another failure. Contemplating an earlier mess in 1930, Keynes had expressed a very similar feeling: “we have involved ourselves in a colossal muddle, having blundered in the control of a delicate machine, the working of which we do not understand” (Keynes 1930, 1). Unlike Hayek, Keynes and others attributed the eruption of the Great Depression, and especially its persistence through the 1930s not to public powers’ eagerness but to their *failure* to intervene: the government’s incapacity to regulate banks and contain financial speculation, its reluctance to actively sustain demand (Keynes 1953), and the Federal Reserve’s unwillingness to serve as a lender of last resort (Bernanke 2000). And if we look to the present rather than to the past, the defanging of state regulatory power in the areas of banking and derivatives are among the most cited causes of the 2008 Great Recession (Campbell 2010, Krippner 2010, Johnson and Kwak 2010). Importantly, these much more market-friendly policy prescriptions, which might have been more to Hayek’s liking, also came with their own scientific justifications and their own guarantees of academic authority: the reforms were not only cheered by those who accept the efficient market view of finance, but they counted the likes of Larry Summers among their champions. In that sense, they were no less inspired by the “pretense of knowledge” than the Keynesians’ advocacy of fiscal policy in the 1960s, which Hayek condemned so forcefully. In short, economic crises act as revelators of the misguided belief that (quoting Hayek again) “we possess the knowledge and power which enable us to shape the processes of society to our liking” (1989, 7). But this belief (and its critique) can both support very different ideological and policy commitments.

## The truth of the market

Where does this belief and the authority of economics come from? Here, it is useful—perhaps—to consider the origin conditions of modern economic discourse. The “pretense of knowledge” in economics—in other words, the belief that economics can provide clear, complete, effective, and unbiased answers to most social problems—has its roots in liberal political culture, and specifically in the role of the market as a “regime of truth-telling” for politics, to use the term proposed by French historian Michel Foucault. According to Foucault, the rise of liberalism in the eighteenth century was fundamentally about defining the limits that government practice must impose on itself. It is around that time that sovereigns’ concerns shifted from the legitimacy of their political rule (in relation to nature, genealogy or the divine, for instance) to success and failure at governing. From worrying about external limits to their own actions (coming from citizens’ assertion of rights and from other states), governments started worrying about designing internal limits to the logic of government itself—in short, how not to govern too much, and how to govern just enough.

Political economy, which arose at the same time, supplied the principle through which those limits were to be demarcated. By knowing the natural laws of the market, political economy offered a way to tell the truth about the correct limits of governmental practice. Government action was not to be judged primarily in terms of legitimacy or justice, but in terms of whether it was right or wrong. And it is the market that was to provide that truth-test, through the work and voice of political economists. “The market must be that which reveals something like a truth. […] What is discovered at this moment [Foucault means the 18^th^ century], *at once in governmental practice and in reflection on this governmental practice*, is that inasmuch as prices are determined in accordance with the natural mechanism of the market they constitute a standard of truth which enables us to discern which governmental practices are correct and which are erroneous” (Foucault 2010, 32, my emphasis). But even in a world where prices are rigid and do not adjust, government could be mobilized to foster the flexibility that should have been (this is the neoliberal solution) or to adjust its action by working with the rigidities (this is the Keynesian solution). Economists, consequently, have become the guardians and the revelators of this truth, not simply in their own eyes, of course, but in the eyes of everyone, and first and foremost in the eyes of government itself. The dynamics that sustain this truth-telling dimension of the “pretense of knowledge”—the rights and wrongs of government action and, increasingly (I will come back to this), of all actions—are thus not simply internal to the discipline. They are all around it, calling it from within the broader field of practice to “fix” everything from climate change to child learning. The techniques for revealing truth may have evolved (from formal models to precisely administered experiments), but the logic of economists seeing themselves as truth tellers to government power—of the sort: this will or will not work—has remained deeply ingrained. 

## The measuring rod of money

One question, obviously, is: why was the truth of the market so compelling to begin with? What, in other words, gave economics an edge over other forms of expertise? Foucault does not offer an answer to this question. My own response will return to precisely those “measurable magnitudes” that made Friedrich Hayek so skeptical. Others have noted that one of these magnitudes—money—is of special relevance. For instance, Ronald Coase argued in a comparison between economics and its “contiguous disciplines” that “the great advantage which economics has possessed is that it is able to use the measuring rod of money.” This, he went on, “has given a precision to the analysis, and since what is measured by money are important determinants of human behavior in the economic system, the analysis has considerable explanatory power” (1978, 209). Coase himself was taking his cues from Arthur Pigou and beyond him from Alfred Marshall, who in his *Principles of Economics*, had stated that it is “this definite and exact money measurement of the steadiest motives in business life, which has enabled economics far to outrun every other branch of the study of man” (Marshall 2013, 12–13).

Yes, money is *the* commensurating agent *par excellence*. It turns qualities into quantities, or collapses subjective variations and “motives”, which may be incompatible, into objective counts. It is impersonal and non-judgmental, and it also allows differences in value and valuation to be revealed precisely. As such, money may help resolve conflicts. But measurement through money is a double-edged sword. Money comes with its own moral baggage and creates all kinds of paradoxes. It spoils motives and effectively changes values. Also, money is not the only source of precision out there. Demography is a rather precise discipline, too: its measuring rods are counts of births and deaths, marriages and divorces. So perhaps we should look elsewhere. If we agree that the measuring rod of money is an important source of the power of economics, what is it that money does besides offering precision in measurement?

Another equally important reason why power over the measuring rod of money translates into real world power is the fact that *money itself* is such a power. Unlike the other social scientific disciplines, economics comes with a promise: the promise to make money, the promise to save money, the promise to allocate money (a rare resource) in the most efficient manner.^6^ In other words, part of the authority of economists also comes from their association with whoever holds the purse strings. They navigate the most powerful parts of the world, where financial decisions are being made and where political and corporate leaders are being trained. And, I shall add, this association has become increasingly tight over the course of the twentieth century. Business schools, for instance, have gone from being intellectual backwaters staffed with practitioners to becoming scientific powerhouses filled with disciplinary social scientists (with economics PhDs being the largest group) (Fourcade and Khurana 2013).

The consequences of this prosperous social position are not trivial. Let us remember that money is not neutral (Frey 1997). It changes people from within. As their jurisdiction has expanded and diversified, economists as a group have seen their financial fortunes multiply. This is especially striking in the USA, where economics is one of the most lucrative degrees over a person’s lifecycle, both at the undergraduate and graduate levels (Weissmann 2014).^7^ The salaries of academic economists have grown faster than any other arts and sciences discipline, including “hot” subjects like computer science, over the last 30 years, and opportunities for extra-academic income have proliferated.

In some subfields, such as IO or finance, economists frequently “go for the gold” as court experts and highly paid consultants (Zingales 2015, Weyl 2017). Deregulation and crisis have fueled an economic litigation boom, which has been largely hidden from view since most of the economists’ activities as expert witnesses take place below the radar, to help negotiate undisclosed settlements (Mandel 1999). But through these channels, and others (such as the rise of think tanks and polarized news media in the US), economics has been enrolled into much more adversarial forms of knowledge production, jumping into power struggles with both feet. This is perilous. Numbers are a technology of trust (Porter 1995) until everyone can (with enough money) produce their own numbers and they are not.^8^

## The denegation of politics

These institutional entanglements of economists have broader implications. Quite simply, the changing social position of economists reverberates onto their political preferences. In the USA, the highly educated and academics in general tend to be on the left, but economics is among the most conservative disciplines (that is, it has a higher proportion of people voting for the Republican party than other fields) (Gross and Simmons 2007). The effects of institutional location and income gradient are palpable: economists in business schools are more conservative than economists in economics departments, with finance and accounting being the most conservative fields of all (Gross and Simmons 2007, Jelveh et al. 2017).^9^

Most scientists work studiously to distance themselves from politics: such a distance, they realize, is a condition of their professed objectivity. Many state bureaucracies actively require it. Even interest groups need their experts to appear impartial. Social scientists have always had to work extra hard, because they were always more suspect. For instance, the social sciences were excluded from the US National Science Foundation at its creation in 1950 on the grounds that their politics would contaminate “the perceived ethical neutrality” of natural scientists (Gieryn 1999). And thus, they have had to build their strength through scientific and institutional strategies that sought to neutralize or counterbalance their inevitable closeness to political struggles (Lebaron 1997). The construction of ostensibly depoliticized “spaces of objectivity”—dedicated expert agencies, expert commissions, the use of experts in parliamentary hearings—was among the important channels that have sustained the rise of economic knowledge to power around the world. Economics’ heavy reliance on formal models not only keeps laymen (and their politics) at a distance, but also produces an effect of impersonality and objectivity. On the empirical side, a certain blind faith (e.g., Cahuc and Zylberberg 2016) in very narrowly specified experiments (even though their results are often ambivalent, and hard to scale up) projects the same neutralization effect. Finally, the field’s general emphasis on efficiency—a seemingly unbiased criterion of choice—and the devaluation (until very recently) of more controversial distributive questions, also participate in this denegation of politics.

In my own work on the historical development of the economics profession in the USA, the UK, and France, I found that economists were anxious to be perceived as apolitical. A prominent labor economist who had worked closely with unions put it succinctly to me in an interview: “you don’t want people to feel like you’re a party hack.” And yet politics is hard to leave behind. Economic “truths” are not easily produced. There are several reasons for this. Some are methodological, such as the necessarily partial character of distributed knowledge (an argument *à la* Hayek), the changing character of the social world, or the low power of statistical tests (Summers 1986). Others are more subtle. Economists may be united—much more united than other social sciences in their core “style of reasoning” (Hirschman and Popp-Berman 2014)—they are very much disunited when it comes to the details of their scientific practice. Academic politics and politics at large are the main sources of their internal conflicts (Romer 2015).

Using results from an opinion survey of 51 economists on pressing policy issues across seven leading US economics departments (the IGM panel), Gunten et al. (2016) find strong evidence of ideological heterogeneity on a subset of these issues: in particular, they show that the institutional saltwater/freshwater divide is very real, and that it is aligned with a pro-state/pro-market divide.^10^ In a recent working paper, Jelveh et al. (2017) correlate economists’ observed political behavior (in the form of campaign contributions and petition signing in the US) with machine learning-derived phrases from their academic articles. They show that the linguistic and institutional universe of economists depends heavily on their political persuasion. But does this affect the *outcomes* of their research? Using the text associations, Jelveh, Kogut, and Naidu are able to “predict the ideology of any economist not in [the] original sample by feeding his or her body of work into [their] algorithm” (2014). The result, again, is that left- and right-leaning economists are reliably associated with different research fields, departments, and methodologies. Most remarkably, empirical work offers no redemption: the authors find a significant correlation between the linguistically derived ideology predictor and the magnitudes of policy-relevant elasticities (wage, income, tax) that authors reported in academic publications.^11^ They conclude: “in other words, a left-leaning economist is more likely to report numerical results aligned with liberal ideology (and the same is true for right-leaning economists and conservative ideology)” (2014).

This, of course, comes as no surprise to sociologists of science, who are familiar with the concept that even the most seemingly solid of sciences remains a social enterprise. (In *Knowledge and Social Imagery* (1976), for instance, David Bloor shows that even mathematics displays numerous variations that can be attributed to sociological causes.)^12^ To go back to the Jelweh et al. study, this does not necessarily mean that people manipulate their results in order to match their ideological priors, but rather that the technical and analytical infrastructures they built are fickle enough that they end up measuring and revealing different aspects of the social and economic world. For instance, we know that the way economists think about economic processes, the kinds of questions they worry about, how they assume and perform their authority and social roles are all strongly shaped by their social and intellectual trajectory, and by the national context in which they operate (Lebaron 2000, Frey and Eichenberger 1993, Fourcade 2009). Here in Europe, we are accustomed with the fact that the views of economists vary in important ways across countries: Southern European macroeconomists, for instance, are noticeability less conservative than Northern European ones. These differences have a long history and are not easily erased: in their book on *The Euro and the Battle of Economic Ideas*, Brunnermeier et al. (2016) spend a chapter detailing the divergence between the French and German economic philosophies, for instance, which have inspired very different responses to the crisis of the Eurozone. The philosophies themselves must be traced back to each country’s path into modernity, its specific economic experiences (the still vivid mark of hyperinflation in Germany for instance), its broader intellectual history, and the modes of institutionalization of economic knowledge in the worlds of education, policy and business.

## Creative destruction

These thoughts, which expose the fundamentally contingent and heteronomous nature of economic knowledge, are sobering, perhaps. Paradoxically, I do not think that they necessarily bode ill for the discipline. First, we should recognize that what Michael Reay (2012) calls the “flexible unity” of economics is a fundamental component of its strength. On the one hand is a fairly united “way of looking at the world” (Coase 1978, 210) and an eminently recognizable style of reasoning, which is applicable across a broad range of domains: in that sense, economics is a truly generalistic form of expertise, defined by its techniques and epistemological processes rather than by its core beliefs about the way the world works. In fact, what we call the mainstream has been malleable enough to incorporate waves of peripheral (and once rejected) ideas and concepts (think: price rigidities into real business cycle models, increasing returns into growth theory, non-rational behavior). As a result, the core has become multiple and fragmented, but it can still legitimately claim to hold up through, rather than against, this fragmentation. As French regulationist economist Robert Boyer (2016) has recently suggested, this is a paradoxical world in which the respective “truths” of Eugene Fama and Robert Shiller can both legitimately exist, and where the very multi-vocality of the field is actually the mechanism that fosters its resilience.

Connected to this is the great operational plasticity of economics in practice, which accommodates a wide range of ideological positions and allows the field to generate its own social demand. Economics is a revolutionary force. It transforms itself by transforming the world. More than the rise of economists as persons, it is the expansion of economic technologies, skills, language and modes of calculation everywhere—economics “in the wild” as sociologist Michel Callon puts it—which constitutes a definite feature of modern culture and the real source of economists’ worldly power. But as economic devices become embedded in real-world markets and institutions (Callon 2007, Hirschman and Popp-Berman 2014), they reconfigure social and economic relations and set in motion new dynamics and strategies that, in turn, become objects for the exercise of economic knowledge and the “fix it” activities of economists. It is a bit as if the development of medicine also generated new pathologies in its efforts to treat existing pathologies (and of course this is partly true for medicine: think about the adaptability of viruses, or the population’s immunity to antibiotics). Is it what Hayek had in mind when he talked about the pretense of knowledge? Perhaps. In the end, the economists’ willingness to intervene may be among the forces that fuel, rather than diminish, the radical indeterminacy and ever changing character of the social world. But this indeterminacy, in turn, plays a role in the intellectual dynamics of economics itself. It becomes the *pretext* upon which new pretenses are built, aiming at the further, if often self-contradictory, refinement of a knowledge that is never, and will probably never be achieved for certain.

## Footnotes

1 Chandler’s historical work provided a useful foil, which sociologists used ad nauseam to debunk the generic argument that “certain institutions exist because they are efficient solutions to various problems in the economy” (Granovetter and Swedberg 2011). A similar critique, rooted in detailed empirical analyses of how firms work, was leveraged against the new institutionalist theory that hierarchical structures arise as efficient responses to problems of transaction costs and opportunism (Granovetter 1985). And nothing compares with the feeling of jubilation that came from exposing the crudeness of Gary Becker’s view of human action, where preferences are exogenously given and “de gustibus non est. disputandum.” (Stigler and Becker 1977).

2 Whatever they thought, the nervous and often negative attention economics received from its sister disciplines did not make it more likely to engage with them. These imbalances have been structuring the social sciences for a long time, and they were certainly there when I entered graduate school in sociology back in the early 1990s. In 2000, 81% of the citations of economics journals went to themselves (the corresponding numbers are in the 50% for sociology, political science, etc.). My own examination (with co-authors Etienne Ollion and Yann Algan) of the structure of interdisciplinary citations in the social sciences in the 2000s revealed profound asymmetries. Between 2000 and 2009, articles in the *American Political Science Review* cited the top 25 economics journals more than five times as often as the articles in the *American Economic Review* cited the top 25 political science journals. The asymmetry was even starker with regard to the *American Sociological Review*. While only 2.3% of the sociologists’ citations went to their economics colleagues, just 0.3% of economists’ citations went to sociologists (again only taking into account the top 25 journals in each discipline), almost 8 times less.

3 The Harvard Economic Service was reorganized as the Harvard Economic Society in 1927, in an effort to sever its relationship to the university.

4 Caballero continues: “We are digging ourselves, one step at a time, deeper and deeper into a Fantasyland, with economic agents who can solve richer and richer stochastic general equilibrium problems containing all sorts of frictions. Because the “progress” is gradual, we do not seem to notice as we accept what are increasingly absurd behavioral conventions and stretch the intelligence and information of underlying economic agents to levels that render them unrecognizable” (2010, 90).

5 For instance, Hayek might have been horrified by Cahuc and Zylberberg's forceful extolling of experimentalism as to path to scientific truth in economics (2016).

6 Let us remember that the discourse of economics in the classical age was entirely focused on the analysis of wealth (Foucault 1994).

7 “Up until about the 57th percentile, engineers make the most. But then the earnings curve for economics grads basically goes parabolic. At the 95th percentile, they can expect to earn more than $3 million more during their lifetime than an engineering grad” (Weissmann 2014).

8 Another consequence is that it is often as members of a privileged class that economists now confront the public (where their privilege is measured not simply by education and social origins, but increasingly by personal income and wealth). Also, inequalities have increased within the economics profession itself, too. The higher monetary valuation of certain economists (and fields within economics) might also create or reinforce beliefs that these inequalities simply reflect an objective hierarchy of knowledge (Fourcade, Ollion & Algan 2015).

9 There is some evidence that—perhaps due to the economic and institutional transformations I just referenced (rising economic fortunes, the rise of business schools)—the economics profession as a whole has experienced a slight shift to the right since the 1970s. Also, there is substantial evidence that laymen exposed to economic reasoning display more selfishness and are more likely to emphasize efficiency rather than equity (Fisman, Kariv and Markovis unpublished manuscript). Professional judges exposed to economics tend to give harsher sentences (Ash, Chen and Naidu 2017).

10 The authors reject the consensus + noise null hypothesis.

11 The authors mitigate the possibility of reverse causation by using only ideology estimated from papers published before the paper containing the reported ideology/elasticities.

12 The sources of variation in mathematics are: (1) the “cognitive style,” (2) the “framework of associations, relationships, uses, analogies, and metaphysical implications attributed to mathematics,” (3) “the meanings attached to computations and symbolic manipulations,” and (4) “the type of reasoning which is held to prove a conclusion” (Bloor 1976, 110).
